# The role of community health workers in non-communicable diseases in Cape Town, South Africa: descriptive exploratory qualitative study

**DOI:** 10.1186/s12875-024-02424-2

**Published:** 2024-05-21

**Authors:** Lize-Marie Doresha, Williams, Robert Mash

**Affiliations:** Division of Family Medicine and Primary Care, Stellenbosch University, Cape Town, South Africa

**Keywords:** Community health workers, Primary health care, Primary care, Non-communicable diseases, Chronic diseases, South africa

## Abstract

**Background:**

Community health workers (CHW) are an integral part of primary health care re-engineering in South Africa. Cape Town is developing community-orientated primary care, with a central role for CHWs. Their role in human immunodeficiency virus, tuberculosis, maternal and child health has been articulated, but is less clear for non-communicable diseases (NCDs). Non-communicable diseases are now a major contributor to the burden of disease. The aim was to explore the current role of CHWs regarding NCDs in the Eastern sub-district of Cape Town, South Africa.

**Methods:**

An exploratory descriptive qualitative study made use of non-participant observation and qualitative interviews with CHWs, their managers, and nurse coordinators. Data from nine semi-structured interviews and ten observations were analysed with the framework method and Atlas-ti.

**Results:**

The CHWs were embedded in their communities and provided services via support groups, household visits and delivery of medication. They linked people to care with assistance of nurse coordinators. They could also provide physical care in the home. They lacked the ability to counsel people on the risk factors for NCDs and their role in rehabilitation and palliative care was unclear. More nurse coordinators were needed to provide supportive supervision. Inter-sectoral collaboration was weak and hindered CHWs from addressing social issues. More standardised and comprehensive training should equip CHWs for health promotion and disease prevention during household visits. Data collected in the community needed to be analysed, reported on and integrated with data from the primary care facility. This should also contribute to a community diagnosis. Their relationship with facility-based members of the primary health care team needed to be improved. Attention needed to be given to the requirements for and conditions of employment, as well as working hours and remuneration. Some equipment was absent and hindered their services for NCDs.

**Conclusions:**

CHWs have the potential to provide a comprehensive approach to NCDs, but community-orientated primary care needs to be strengthened in many of the key areas to support their activities. In relation to NCDs, they need training in basic and brief behaviour change counselling and risk factors as well as in the areas of rehabilitation and palliative care.

**Supplementary Information:**

The online version contains supplementary material available at 10.1186/s12875-024-02424-2.

## Background

South Africa has a quadruple burden of disease with an increasing contribution from non-communicable diseases (NCDs) that particularly affects poor urbanised communities [1]. Diabetes is now the second leading cause of death in South Africa [2], and the COVID-19 (Corona virus infectious disease-2019) pandemic highlighted the growing problem of diabetes as a major risk factor for mortality [3]. The most recent strategic plan for NCDs recognises five key diseases (cardiovascular, cancer, chronic respiratory, diabetes, and mental health) and five key behavioural risk factors (tobacco use, unhealthy diet, physical inactivity, harmful use of alcohol and air pollution) [1]. The strategy sets a 90-60-50 cascade of goals, similar to the approach used in human immunodeficiency virus (HIV) and tuberculosis (TB). This cascade aims for 90% of people to know their NCD status, 60% of those with an NCD to receive an intervention and 50% of them to be well controlled. Currently there is insufficient national data to measure these goals, but evidence suggests they are not being met. For example, in the Cape Town metropole only 25% of people with diabetes are controlled (HbA1c < 7%) [4].

South Africa is committed to a health system based on high quality primary health care (PHC) [5]. Re-engineering of PHC emphasised the need for district clinical specialist teams, improved school health services and ward-based outreach teams (WBOTs) [5]. The WBOT concept introduced teams of community health workers (CHW) to extend the reach of PHC facilities. The South African Medical Research Council evaluated the approach and found it to be cost-effective with the potential for a substantial return on investment – saving money, while improving health status [6]. In the Western Cape Province these CHW teams were conceptualised within a community-orientated primary care (COPC) approach [7]. In this model, local non-government organisations (NGOs) employed CHWs and professional nurses as team leaders. The plan was for 10–15 CHWs to be led by one professional nurse and each CHW responsible for around 250 households or 1000 people.

Prior to the end of Apartheid, many NGOs had employed CHWs to offer PHC to communities without sufficient formal health services [8]. After 1994, the new government was keen to introduce accessible formal PHC services and many of these NGO-led CHW projects collapsed. The projects that continued often had CHWs that targeted specific diseases or services such as home-based palliative care, HIV or TB. The introduction of WBOTs promoted a more comprehensive role for CHWs that was focused on a geographic community and linked to a PHC facility. In the Western Cape the COPC approach took this even further with a desire to focus on the health needs of the population at risk and not just see CHWs as a form of facility-based outreach [7]. The Metro Health Services (MHS) developed a 10-point COPC implementation framework that was developed in four pilot sites [7].

At the same time the MHS recognised the growing number of people on treatment for NCDs and that their services were becoming overwhelmed. A differentiated model of care was considered, whereby stable and relatively well controlled people with chronic conditions would be managed more in the community [9]. Thus, freeing up clinicians at the facility to focus on the unstable and poorly controlled patients. This model proposed that NGOs develop support or adherence clubs where patients could also collect their medication. The role of CHWs in preventing and managing NCDs became increasingly important, but their exact scope of practice was unclear. Their role in NCDs would also need to be part of a comprehensive scope of practice across the burden of disease.

The aim of this study was to explore the role of CHWs with regard to NCDs in the Eastern sub-district of the MHS in Cape Town, South Africa. Specific objectives included:

- To explore the experiences and expectations of CHWs with regards to their role in NCDs.
- To explore the experiences and expectations of substructure and sub-district level managers with regard to the role of CHWs in NCDs.
- To explore the experiences and expectations of NGOs with regard to the role of CHWs in NCDs.

## Methods

### Study design

The study design was a qualitative exploratory descriptive study that made use of non-participant observation and qualitative interviews with key informants.

### Setting

In 2023, the Eastern subdistrict of Cape Town was projected to have a population of 751,109 extrapolated from the 2011 census [10]. Approximately 80% of the population were dependent on public sector health services and lived in low socio-economic settings, speaking Afrikaans, isiXhosa or English. Helderberg District Hospital provided secondary and emergency services to the community, which is also served by ten PHC facilities. Nomzamo health centre was a pilot site for the implementation of COPC. Masincedane was a local NGO, contracted by the Department of Health, to serve the whole of the subdistrict. It provided an array of services, which included promotion of personal hygiene, client mobility for stroke survivors, dispensing of chronic medication, stoma care and support of TB treatment [11]. For chronic diseases, they organised four support groups for diagnosed patients, where they offered health promotion, monitoring of blood pressure, foot screening, blood glucose testing and the dispensing of medication. CHWs received a four day chronic disease and lifestyle support group training. The CHWs were contracted from 08H00 to 13H00 on weekdays and to be employed were required to have completed grade 9 (matriculation from high school is grade 12). CHWs worked mostly within the community-based support groups and at household level. Some CHWs were based at frail care centres or at health care facilities doing HIV counselling.

### Study population

All CHWs were regarded as equally important key informants and therefore ten were randomly selected from a list provided by the NGO and invited to be interviewed. Five CHWs were interviewed and following initial analysis a further five were interviewed to ensure all key topics were fully explored. CHWs had various years of experience and due to random selection CHWs with different experience had an equal chance of being selected. If new themes were still emerging after these 10 interviews, then further interviews would be considered to ensure data saturation was achieved.

The nurses who coordinated the CHW teams, the NGO managers who were responsible for the performance of CHW teams and the Department of Health’s (DOH) managers who contracted the CHWs’ services also had important perspectives on their roles and what could or should be done for NCDs. One of the more experienced nurse co-ordinators employed by the NGO was selected for interview. In addition, the NGO manager and managers responsible for community-based services at the substructure (Khayelitsha-Eastern) and sub-district (Eastern) levels were invited to be interviewed.

### Data collection

Individual semi-structured interviews were based on interview guides (Supplementary file 1) and explored a number of key topics. The topics were identified from the literature and what was currently recommended in health policy [12–14]. Key topics for CHWs were:

- Their relationship with the community.
- Their perceived role in relation to NCDs and risk factors.
- The strengths and weaknesses of the current services for NCDs.
- Cooperation, referral to and partnership with primary care facilities and staff.
- Pros and cons of their current training in NCDs.
- Their perceptions of the future COPC model for CHWs.

The interview guide with NGO managers or co-ordinators focussed on the same topics as above and in addition explored:

- Inter-sectoral collaboration.
- Relationship with the DOH.
- Monitoring and evaluation of the CHWs and health information systems.

The interview with the DOH managers, focussed on the following topics:

- Relationship with the NGO and CHWs.
- Monitoring and evaluation of the CHWs and health information systems.
- Assessment of their current impact.
- Strengths and weaknesses of the current services for NCDs.
- Perceived roles of the CHWs in relation to NCDs now and in the future.
- Training needs and current training.
- Cost-effectiveness and financial issues.

All interviews were audio recorded and conducted by the principal researcher in English or Afrikaans over a 60 min period. The principal researcher attended training in qualitative interviewing offered by the Division of Family Medicine and Primary Care. An initial pilot interview was conducted with a CHW in order for the supervisor to provide feedback on the interviewing and communication skills. Interviews were held at the participants’ place of work or at a mutually convenient venue (e.g. the home of the CHW).

The researcher accompanied CHWs in their daily work as a non-participant observer to observe how they perform household visits, facilitated the four support groups and other daily activities. Detailed field notes were made during observations or immediately afterwards. The focus of the observations was the activities of the CHW in relation to NCDs.

### Data analysis

Audiotapes of the interviews were transcribed verbatim by a professional transcription service and field notes from the observations were also included as qualitative data. Transcripts were checked for accuracy against the audiotapes prior to analysis and corrected if necessary. Thematic analysis used the framework method and as well as Atlas-ti software (version 8) [15]. The analysis was inductive and had the following steps:

- Familiarisation: Potential codes were identified by reading the transcripts and field notes from observations.
- Coding index: Codes were finalised and organised into categories, while keeping the objectives of the study in mind.
- Coding: All data sources were coded.
- Charting: All the data derived from the same code was charted together.
- Interpretation: The charts were interpreted, looking for key themes and relationships between themes.

The trustworthiness of the analysis was improved by triangulating data from different methods, (interviews and observations) and from different data sources (CHWs and managers) during the interpretation of the data. The supervisor provided peer review of the analysis process when the coding index was developed and when the data was interpreted and reported.

DW was a registrar in family medicine at the local district hospital. She was trained in qualitative methods by Stellenbosch University and supervised by RM. She had no prior relationship with the participants and while interested in the topic, had no strong views or beliefs regarding the roles of CHWs in NCDs. This enabled her to interview and observe the CHWs from a neutral and open perspective.

## Results

### Profile of respondents

A total of nine interviews were performed, which included six CHWs, the NGO manager, an NGO nurse coordinator and the substructure manager of community-based services (Table 1). In addition, 10 observations were made of the CHWs during their normal working hours on 10 separate days. The researcher accompanied CHWs employed by Masincedane as they made home visits and conducted support groups.

**Table 1:** Profile of the respondents

Interview	Title	Gender	Years of experience as a CHW
Interviewee 1	District manager	Female	-
Interviewee 2	NGO manager	Male	-
Interviewee 3	NGO nurse coordinator	Female	-
Interviewee 4	CHW1	Female	< 5years
Interviewee 5	CHW2	Female	3–5 years
Interviewee 6	CHW 3	Female	5 years
Interviewee 7	CHW 4	Female	> 5years
Interviewee 8	CHW 5	Female	> 5years
Interviewee 9	CHW 6	Female	> 5years

### Overview of themes

The findings are presented as nine themes:

- Theme 1: The relationship of CHWs with their community.
- Theme 2: The relationship of the NGO with the DOH.
- Theme 3: The role of CHWs in NCD and risk factor management.
- Theme 4: Relationship with and referrals between CHWs and their primary care facility.
- Theme 5: Training of CHWs in NCDs.
- Theme 6: Inter-sectoral collaboration.
- Theme 7: Financial issues pertaining to employment and training of CHWs.
- Theme 8: Support and supervision of CHWs.
- Theme 9: Monitoring and evaluation of CHWs and the health information system.

**Theme 1: Relationship of community health workers with their community.**

CHWs were selected to work in the same community where they lived, had been working for many years and were well known in the community. Their relationship with the community was characterised by trust that was built up over time. Community members had to trust them to not disclose their confidential or private information and to give them access to their personal space (e.g. to help them wash). This continuity was also important for community members to trust the advice and information received from CHWs:

*“Sometimes we go and we do baths with them (the patients) and assist them with self-care and personal hygiene and I can see that they feel uncomfortable especially if they are not familiar with and you know your body is a very private thing. I think (they) are afraid that we (community health workers) will talk to other people about them. So it is very important for the community members to earn your trust.” (CHW 5)*.

CHWs and the NGO manager felt that their work was meaningful and rewarding and their contribution was significant to the communities they served:

*“Well we have been around for a long time and we are based in the community and the community health workers live in the community. So we have got a good reputation and we work closely with the community leaders and even the residents. They have come to know us and they have come to know what we are doing and what we are standing for. So we are getting where we actually want to be, with the community people as well as the leaders and all the other stakeholders.” (NGO manager)*.

**Theme 2: The relationship of the NGO with the Department of Health.**

The services rendered by the NGO were defined by the DOH in a service level agreement. Services could be an adjunct to services in the primary care facilities that targeted specific patients or could be additional services that targeted the whole population at risk:

*“The relationship with our NGOs’ in the Eastern sub-district is a professional relationship on behalf of where they are doing a service to the community, on behalf of Department of Health. So the relationship is a service rendering, we give them an outline service agreement were we exactly say can you please do these duties for us and we measure in monitoring and evaluating on a quarterly bases to see how the service was rendering and that is the relationship. It’s a nurse driven relationship that is more about promotion and prevention and that is the kind of relationship that we have at the moment.” (District Manager)*.

The advantage of the NGO being linked to the DOH was that funding and training was provided to CHWs and aligned to health targets that the department wanted to reach. The DOH monitored and evaluated the services provided by CHWs on a quarterly basis:

*“Department of Health is funding us, so they, they are paying for the nurses and the community health workers, so it is a partnership and you know it’s a very close partnership, we, they do a lot of training and we are invited to that training. We interact with the Department of Health in the clinics, the hospital and at departmental level.” (NGO manager)*.

**Theme 3: The role of CHWs in NCD and risk factor management.**

CHWs worked from different sites within the community including households, community halls, churches and the primary care facility. Their scope of practice was comprehensive from health promotion to home-based care although they were delivering a service that was more orientated towards health promotion and disease prevention.

Traditionally CHWs performed home based nursing care and assistance with basic physical care needs such as feeding, bathing, dressing, wound and pressure care, but their recent duties entailed household assessments. This was a task whereby CHWs systematically assessed the households that they were responsible for within the community. Information from the household assessment form was meant to give the DOH an idea of the health status of the residents in a particular area:

*“We evaluate their clinic cards or the road to health charts of children, note all the illnesses people have whether you have asthma, hypertension or diabetes, if you are smoking, if the children’s immunizations are up to date, note the condition of the home. You are then meant to go back to that household and see if there is an improvement, measure their (the patients) glucose and blood pressure and refer to the clinic if there are no improvements.”* (CHW4).

The household visits identified individuals at risk who might require referral to health or social services. CHWs reported findings to their coordinator who then decided on appropriate action, such as referral. Needs that were identified included services from the South Africa Social Security Agency for social grants as well as access to sanitation and electricity, referral to old-age homes, testing for TB or HIV, incomplete immunisations or vitamin A prophylaxis, need for family planning or antenatal care or just information on healthy lifestyle:

*“The household form identifies demographic information about the family, CHWs use the opportunity to see whether the child is immunised, and vitamin A is up to date, this is how we also then refer back to the clinic.” (NGO manager)*.

CHWs provided health promotion to people at risk of NCDs to reduce risky behaviour. This could take the form of health talks in the clinic, support groups or counselling in the home. The communication style was often didactic as they had not received training in behaviour change counselling. The NGO manager iterated the need to integrate behaviour change counselling in the training of CHWs because behaviour change is not guaranteed with health education only:

*“I talked to them (the patients) and explain the reasons to quit smoking. I’m not telling them to stop at once but to start by smoking one less on a daily basis. The patient would adhere to my advice and when I see her again she will say; ‘can you see I’m not short of breath today because I didn’t smoke so much, or I didn’t eat so much’ because I told her she must curb the salty food as well and told me that she had curbed it. Well that influence you have on them when they see you they immediately start telling, you gave me this advice and I have followed it. Sometimes we have pamphlets and then we give them pamphlets regularly.” (CHW 6)*.

CHWs also prevented disease through screening for high blood glucose and blood pressure (BP):

*“We do their BPs and we take their sugar levels. If it’s a bit high, we refer them. We do have letters whereby we refer, we fill it out and then we send them with the letter to the clinic where the clinic staff will take it up with them.” (CHW 5)*.

CHWs provided assistance to people with diagnosed diseases through support groups and household visits to enable self-management and adherence to treatment. Lifestyle modification and health promotion (e.g. dietary advice), as well as diabetic foot care (e.g. cutting of toenails, removal of callus) and identifying patients with uncontrolled diabetes or hypertension, were all part of the daily activities observed. Challenges due to lack of equipment or supplies such as not having various BP cuff sizes, scales or not being trained in brief behaviour change counselling were a barrier in addressing the health needs of patients and referral to primary health facilities:

*“They have a weekly group of elderly people or people who are coming to collect their medication at the group. Obviously we need to give some service, quick help, prevention, promotion, do the blood pressure, do the sugar testing and ABC in diet, dietary things and are you still okay, are you eating okay, you know all that kind of things that is what we do at the group…for chronic disease of lifestyle and then we offer also foot care and to look at their feet and do the foot care, and the cutting of the nails.” (NGO manager)*.

CHWs disseminated pre-packaged medication to stable patients at a monthly support group instead of the pharmacy. This reduced the workload on the clinic and waiting times for the patients. Their role at the community health centres was to liaise with the pharmacists to collect chronic medication for support group patients, give feedback on which patients collected their medication and to clarify any uncertainties related to medication. Although patients were meant to be stable and not needing regular clinic visits, CHWs often found patients with uncontrolled NCDs and referred them back to the primary care facilities.

CHWs also assisted primary care facilities with patients who had defaulted medication or missed their appointments for doctor’s visits or medication collection. Those patients were either picked up via household assessment or support group visits. However, the clinics did not make use of CHWs to visit patients with NCDs identified by the clinic itself:

*“If they pick up somebody with an unacceptable BMI (body mass index) or blood pressure they (CHWs) can fill in a referral form to the clinic and if we get it right there will also be feedback that they have been to the clinic.” (Nurse coordinator)*.

CHWs might also help people at home with complications of their NCDs, such as stroke. They might also provide basic home-based care to people with end-stage NCDs. Rehabilitation and support was given to stroke survivors either at household level or at support groups through partnership with other NGOs within the community.

### Theme 4: Relationship and referrals between CHWs and primary care facility

The lack of a standardised referral process between the CHWs and their different primary care facilities was a barrier to helping people with NCDs. At some facilities the CHWs had easier access, due to good relationships with primary care providers, in comparison to facilities where the role of CHWs was not clearly understood or where no consensus was reached on the appropriate referral pathway. The team dynamics between the CHWs and clinical nurse practitioners could either be a barrier or advantage. Facility-based and community-based services worked in parallel in the same community with liaison, but not full teamwork. CHWs also struggled with their roles and scope of work, perceiving tensions between community needs and expectations and the services they were equipped and authorized to deliver:

*“They will just ask the patient who sent you here and then they will say it’s the nurses (referring to CHWs) in the community and then they will disagree with your referral. It makes me feel that some of the staff they are undermining the care, us as community workers.” (CHW 4)*.

CHWs felt that in some instances, nurses at the facilities had misconceptions regarding their roles and responsibilities and this led to tension in their relationship:

*“Once you send them (the patient) or make a referral you are told by the clinic you are just a community worker, you can’t just do this and for us it challenging and you feel helpless because the family ask you what your job really is.” (CHW 6)*.

In some cases, the primary care facility lacked comprehensive primary care services and could not adequately support the CHWs when patients were referred:

*“In GB clinic, there is no doctor here and first of all when they come there to the clinic, they then tell the (patients) they (the primary health care facility) don’t have a doctor so they can’t treat them, even if they are hypertensive, they can’t monitor them, they must go to a clinic where there is a doctor. So for them to go there, sometimes they don’t have money, they don’t have transport, so it’s difficult for them and they end up not going.” (CHW 2)*.

The NGO developed a partnership with the district hospital and built relationships with local clinics to strengthen referral pathways. Referrals to and from the NGO; came from hospitals, intermediate care and primary care facilities. The referrals from community-based to facility-based services, were to achieve further control and optimisation of NCDs and included people with uncontrolled hypertension and diabetes. These patients were usually identified during household assessments and support group visits:

*“And at first we couldn’t just go in and, and discuss our things, we had to wait, but if we make an appointment now, we do arrange meetings and we can discuss our problems and stuff. If they have a problem they will call us also in but the referral has improved considerably.” (Nurse Coordinator)*.

People with end-stage NCDs that required palliative care were referred to the NGO for assistance with personal hygiene, and prevention and treatment of pressure sores:

*“Well as you can see we are doing the chronic care outside, taking care of the very frail people, where they are doing full body washes, pressure and wound care.” (Nurse Coordinator)*.

Socio-economic problems such as lack of transport and financial issues also prevented patients from attending the nearest health facilities.

### Theme 5: Training of CHWs in NCDs

Each CHW had basic training in home-based care from the NGO through classroom as well as workplace-based training. Non-communicable diseases were part of their curriculum and the NGO used allied health professionals such as physiotherapists and dieticians to teach about chronic diseases, which included rehabilitation for stroke survivors or amputees and lifestyle modification:

*“In their training session we get the physiotherapist in to show them the correct way of lifting people out of the bed into a wheelchair and back. CHWs get trained about the size of the wheelchair and how that should be corrected towards the body of the patient.” (Nurse coordinator)*.

CHWs expressed their need for more in service training especially when they encountered challenges on home visits or at support groups because not all topics were covered by their curriculum:

*“Some of my patients are on insulin and not all CHW are trained to administer insulin, you have to go for training to be able to teach your patient.” (CHW6)*.

The DOH were also involved with the training of CHWs and the training was based on the perceived needs within the community, however the training was done on an ad hoc basis:

*“Training is available, it’s cost effective because it’s us and we normally source people like Medecins Sans Frontieres (MSF) that will do HIV and TB training at their expense. Financial issues, obviously state pays for it, but if there is formal training like the EPWP (Expanded Public Works Program) it can be costly and their course is four years.” (District Manager)*.

The sub-district would then do a performance assessment to see whether CHWs were able to perform the duties for which they were trained in:

*“So that is a consistently monitoring, evaluation because the community based platform it is evolving we have go back because every time they just add to the workload. So when it comes to the monitoring and evaluation we need to see constantly is it relevant, can they do it, are they trained to do it if they need to get some training to do a specific task.” (District Manager)*.

The training specifically for NCDs included how to run a support group and distribute medication, give dietary advice and how to assist patients with complications of chronic diseases, such as wound care. For stroke survivors or amputees they assisted with self-care and hygiene as well as mobilization. Social issues were a challenge for CHWs because their training did not equip them to have an approach to what they encountered:

*“We do, we do get some workshops from the department of health where we can attend. They also have to do projects on patients with chronic diseases where they do a practical on patients living with NCDs who resides within the community. They also observe what we (Nurse coordinators and other trained CHWs) are doing in practice.” (Nurse Coordinator)*.

Different educational backgrounds were a challenge when training CHWs. One particular CHW had done training through a college whilst the minimum requirement at this particular NGO was a grade nine education (the end of high school is grade 12). The different educational backgrounds appeared to influence their ability to achieve the learning outcomes and perform their roles:

*“CHWs are lay workers so you can put ten community health worker in a class, five of them won’t understand well and you have to go back and check ‘do you understand what I am trying to say’.” (District Manager)*

Patients needing rehabilitation were a challenge for CHWs:

*“Say for instance we get a patient that is like a stroke patient and that patient didn’t get down-referred by the hospital, it was through a support group, then we have to do all the exercises, we have to make sure that the BP and everything is right. So I think there is a big need for us to have that proper training so that we know what to do and when to refer them.” (CHW* 5)

### Theme 6: Inter-sectoral collaboration

The NGO reported on a number of important inter-sectoral and stakeholder collaborations and appeared open to pursue and consolidate these relationships. They noted that other NGOs in the area could be useful allies in addressing health issues, even if they were funded from other sources. Primary health care services were not completely integrated due to dual services from the DOH (provincial government) and City of Cape Town (local government) despite serving within the same catchment areas:

*“I’m working with funded NGOs, but your non-funded NGOs (not funded by the DOH) is just as important to pull them also in because obviously sometimes they are working in areas that you don’t even know about or they know things that you are not aware of and they get for example, funding from City of Cape Town and then you can just pull them all together.” (District Manager)*.

Relationships with key role players such as religious leaders and ward councillors within the community had also benefited the NGO by having church buildings and other community halls available for support groups close to patients’ homes:

*“I would say we engage with the community leaders to do out-reaches, it’s all about recognition and also they can spread the word and go to the other forums, we have a relationship with this in the community because you are in the community, it’s like part of their community profile. So that is one of the strengths that we can use for our services in the community.” (District Manager)*.

The CHWs benefited from these relationships and knowledge of local stakeholders by referring people to these additional services, for example, environmental health officers:

*“We have got a partnership, for example where, if we have got referral we can do it to social services, we can do it to environmental health, we are active in an organisation called multi-sectoral action team which is a collective of NPOs and it’s a service we offer in a sense.” (NGO manager)*.

Some key collaborations were difficult to develop, particularly with the Department of Social Development and the police:

*“DSD (Department of Social Development) is one of our main co-partners but we just never get them around the table to plan together as well as collaboration with SAPS (South African Police Service) in terms of the safety of our CHWs to see that they are protected if they [are] entering into an area.” (District Manager)*.

Other collaborations with private, alternative or complimentary practitioners were still to be explored:

*“Also your private sector, I am coming with the option if you go and sell the idea that instead of clients going for family planning I can come monthly and do your family planning and your pap-smear so the woman don’t need to go to the clinic and for a full day.” (District Manager)*.

### Theme 7: Financial issues pertaining to employment

The challenges that CHWs faced in delivering an integrated service were influenced by their working hours, educational and career progression within their field. CHWs were paid below minimum wages, which led to lack of job satisfaction and little opportunity for professional development. Currently CHWs at this particular NGO were receiving a stipend determined by the DOH:

*“It’s very little money for the community health workers. I believe they are going from next year they are going to pay them the minimum wage and there are financial issues pertaining things.” (District Manager*).

The District Manager recognised that due to the progress in the community based platform it was vital that salaries paid to CHW were cost effective in terms of the services delivered by CHWs:

*“We need to evaluate how much they (CHWs) can do in a given time because they are working half day and we [are] giving them a specific package and that is our role to see what we ask from them is it feasible for them to do in a given time. So that is a consistently monitoring, evaluation because the community based platform it is evolving and the workload of the CHW are increasing and we have to evaluate whether the work of CHW are relevant, can the CHWs do it, are they trained to do it, to see where more training is needed to do a specific task.” (District Manager)*.

### Theme 8: Support and supervision of community health workers

They were supervised by nurses who were also employed by the NGO. Their role entailed in-service training, accompanying CHWs on household visits, analysing and reporting on household data, monitoring CHW performance, assessing the need for referral and assisting clinically with complicated cases or housebound individuals who did not have easy access to primary care facilities. Nurse coordinators looked after groups of CHWs and indicated that due to competing demands they were not able to go with CHWs on a daily basis. Work plans and challenges were discussed at a gathering point within the community prior to each work day:

*“Okay they are supervised by us as the coordinators, in the morning we get together and we, we have our work plan set out for the week or sometimes it changes and then we have it for that day, but actually it’s been worked out, what we are going to do the next day. We get together and we discuss and we also give in-service training. Uhm while they (CHWs) are there and they have a problem we will discuss it if they don’t know how to do it, we (nurses) will actually go with them and we will supervise them while they are busy or otherwise if they still seem to be unsure, we will do that ourselves and then they can learn from the way that we are doing things (referring to practical tasks and activities).” (Nurse coordinator)*.

### Theme 9: Monitoring, evaluation and the health information system

Data collected on paper from household assessments were not captured and analysed, but stored at the NGO. The data that was captured provided information on performance of CHWs for provincial and national DOH and were not used to make a community diagnosis or identify health needs. Data were not integrated into the PHC information systems and therefore not used for the purpose of health-related decision making and planning:

*“How we measure it is obviously we say we want you to do ten per month (household assessments). Now out of that household assessment form that I call a census form you can gather a lot of information. You can measure a lot of things, you can pick up for instance the main house consist of five informal houses, each with their own household. So CHWs must register each household on a different form due to the informal settlements residing on the same yard. You get different disease profiles from each household. We want to have an idea what is happening in the community so we can plan accordingly. Say for instance here in block A has a lot of diabetes or hypertension or then we know we must have outreach pertaining to the disease profile. We informally measured what the impact is of community health workers.” (District Coordinator)*.

A m-health solution, called Catch and Match, was being piloted amongst the CHWs. There was an attempt through the provincial data centre to link this data collected from CHWs with health facility data via a single patient viewer system. This was an opportunity to analyse the health status of the community. At the time of this research the district manager expressed concerns that CHWs might neglect their regular work and only focus on capturing data on their cell phones. The CHWs had limited understanding of the Catch and Match system and adoption of new technology was not easy:

*“Okay, all of these things normally start out with pilots and it takes long to change the mind of the community health worker because it’s almost like you are trained to do that and that’s it. So that is my, my perception about it and that is their perception too, we have lots of challenges with that.” (District Manager)*.

## Discussion

The CHWs had several roles and performed a range of health care activities for people with NCDs, from health promotion, disease prevention, to adherence and treatment support, assistance with rehabilitation and palliative care. However, there was a need for further training as they were unprepared for many of the health and social challenges that they encountered. The key findings are summarised in Table 2 and categorised into strengths, weaknesses, opportunities and threats.

**Table 2:** Summary of key findings

**Strengths** • Embedded in the culture of the community and good relationships with community members • Support groups promoted self-management and lifestyle change, checked control of NCDs (e.g. blood pressure, blood glucose and weight) and referred to the clinic if necessary. • Household assessments identified people with NCDs and smokers. Provided advice, such as smoking cessation, and checked on adherence. • Disseminated pre-packaged medication to patients at support groups and sometimes via home visits. • Assisted patients to make new clinic appointments when they had defaulted. Nurse coordinators assisted with coordination of care between facilities and CHWs. • Home based nursing care and assistance with basic physical care needs such as feeding, bathing, dressing, wound and pressure care. • Professional nurses (nurse coordinators), supervised and supported CHWs, for example by doing home visits for complicated cases where they assessed the patient and advised on management.	**Weaknesses** • Reducing the risk factors for NCDs required more training in behaviour change counselling, knowledge of the risk factors and who can assist further in the community. • Insufficient numbers of nurse coordinators to adequately supervise and support CHWs at support groups. • Inter-sectoral collaboration was weak and this particularly hindered the ability of CHWs to access social services and help with social issues.
**Opportunities** • A multi-sectoral action team already existed including the DOH. • There was an opportunity for more standardised and comprehensive training through the DOH once the training programme was finalised. • There was an opportunity to do more, especially during the household assessment, and to screen for people at risk of NCDs e.g. cardiovascular risk score, screening for substance or alcohol abuse. • There was an opportunity to evaluate the data gathered at the household assessment more accurately as part of a community diagnosis, to plan interventions and to integrate into the broader health information system. • There was an opportunity to enhance the roles of CHWs in rehabilitation processes (e.g. support of stroke survivors) and palliative care (e.g. pain management)	**Threats** • The work of the CHWs was threatened by a poor relationship with the facility-based members of the PHC team. Poor coordination between the facility and community based teams with a lack of respect, support and collaboration. • Inadequate or lack of equipment to perform their duties in the support groups e.g. scales, various BP cuff sizes, patient information leaflets. • Lack of clarity on the scope of practice of the CHWs, particularly in relation to NCDs. • Salaries for CHWs and number of contracting hours were low for the duties expected to perform • CHWs were selected at too low educational levels to adequately perform all of their tasks.

The CHWs have an integral role in the National Development Plan, which envisages a comprehensive role for CHWs in NCDs; including health promotion, disease prevention, palliative and rehabilitation care [16]. There is also an expectation that CHWs will play a pivotal role in the provincial strategy to reduce the prevalence of NCDs [13]. CHWs can be cost-effective at screening households for NCDs and providing basic primary care [17]. The findings of the study showed that CHWs undertook a wide range of health care activities related to NCDs, but these were somewhat ad hoc and not well defined in policy. Studies elsewhere also highlight the need for clearer and more specific policy and guidelines on the role of CHWs in NCDs [18–20].

Subsequently the Metro Health Services agreed on a scope of practice with the following generic key performance areas: community entry, identification of health assets and risks in households and communities, acting for and empowering people, contributing to community and stakeholder engagement, and building relationships within the PHC team. Acting and empowering included health promotion, screening for disease, physical care, adherence support, rehabilitation and palliative care. The specific tasks related to NCDs, however, were not defined. In other settings the tasks required of CHWs for NCDs include screening for disease, provisional diagnosis, health education and counselling, dispensing of medication and referral to healthcare facilities [19, 21].

The study perceived a lack of skills to perform many of the required tasks and this can be attributed to a lack of and poor quality of training as well as a lack of sufficient supervision. There are risks with task shifting to CHWs without sufficient training as this can compromise quality of care, patient safety and erode community confidence in CHWs [18, 22]. Several studies have also commented on the need for better training of CHWs to tackle NCDs [18, 19, 22, 23]. This highlights the need to have standardized training that is tailored to the context in which CHWs are working and addresses the local burden of NCDs [24, 25].

The DOH envisage that CHWs will address the risk factors for chronic diseases through lifestyle modification strategies aimed at tobacco smoking cessation, physical activity, healthy diet and alcohol reduction [1]. The findings suggest that risk factors for NCDs were poorly addressed and this also highlighted the need for training in communication skills for basic and brief behaviour change conversations [26]. CHWs with the self-efficacy to make effective lifestyle choices for themselves are more likely to be effective role models and to engage with NCD prevention [27].

CHWs with less educational foundation and less experience had difficulty conducting health talks and explaining nutritional concepts. Key issues are the selection of CHWs with a sufficient educational level, a standardised training programme and alignment of the programmatic learning outcomes with the roles expected of CHWs in policy [24, 25]. The Metro Health Services later made a decision to only appoint CHWs who had completed high school.

The roles of CHWs in rehabilitation and palliative care are not well defined in policy although this forms part of the National Development Plan for 2030 [16]. CHWs expressed the need for further training, despite having some prior training in rehabilitation, as they felt unequipped for this role. A recent study developed a training programme for CHWs to assist caregivers of stroke survivors during household visits [28]. CHWs were often the only part of the health services engaging stroke survivors, but without any training in how to assist [29]. Hospital stays were very short and formal rehabilitation was difficult to access [30]. A similar situation is seen with palliative care, where CHWs tread a path between their official scope of practice and training, and their awareness of palliative care needs in the community [31]. In a context where formal palliative care services are scanty and difficult to access, CHWs may be the most tangible part of the health service. The nurse coordinators may have a particularly important role in home-based palliative care [31].

The National DOH recommended that CHW teams should consist of six to ten CHWs supervised by a nurse coordinator (professional nurse) to ensure adequate coverage of the defined population [32]. In this study context, nurse coordinators supervised groups of CHWs that exceeded the 1:10 ratio. The Metro Health Services recognised that nurse coordinators must fulfil a clinical and not just an administrative role. Problems with supportive supervision have been noted elsewhere [12, 19, 21], and in this context were often due to nurses from primary care facilities being asked to supervise CHWs on top of their existing duties.

Inter-sectoral collaboration forms a pivotal part of the COPC approach [7]. In this study the NGO identified important stakeholders that could provide venues as well as services for people identified by CHWs. At the time of the study, collaboration between primary care services from local and provincial government, in the same community, required improvement. The NGO was part of a multi-sectoral team along with the DOH, but developing relationships with some key role players, such as the South African Social Security Agency, was difficult. Weaknesses in inter-sectoral collaboration, particularly between health and social services has also been noted in other parts of South Africa [12].

Outsourcing of certain health services to NGOs was a cost-effective strategy for the DOH, but concerns were raised that this approach might disregard the Labour Relations Amendment Act No 6 of 2014 and inadvertently exploit vulnerable CHWs, who were often paid below the minimum wage [33]. Of note is the implication in the Act that NGO staff can technically be considered employees of the DOH who should receive similar benefits as permanent workers [33]. Adequate remuneration and moving away from volunteerism is supported in the literature [12, 19, 21]. The perspective of the substructure manager and the CHWs were that the workload sometimes exceeded the contracted hours. An excessive workload and competing demands from multiple health programmes are an issue for CHWs [19, 21]. The DOH subsequently increased the number of hours and remuneration, but the need to balance expectations between health programmes persists.

The perceived resistance from facility-based staff to cooperate with the CHWs was attributed to a lack of respect and clear scope of practice. CHWs particularly struggled with referring patients to the primary care facilities. In local studies, CHWs performed better in their collaboration with the HIV and TB programmes, where their role was more clearly defined and appreciated [34]. The implementation of community-based services, that are fragmented and not fully integrated into a well-functioning PHC team, is likely to be less effective [34].

During COVID-19 the value of CHWs was highlighted, particularly their ability to deliver medication at home to people with NCDs and to avoid exposing them to the risks of public transport and health facilities [35]. CHWs were also valuable in community screening and testing, although this was limited by laboratory capacity [36]. Many CHWs also assisted facilities with re-organisation of service delivery [37]. As a result relationships and respect improved in many areas and facilities set up multi-disciplinary team meetings to coordinate care with the CHWs.

The National DOH intends to develop standardised data collection tools for all WBOTs and to integrate with data from primary care facilities [32]. The National DOH developed m-health strategies to assist CHWs to collect data and to capture this data within the broader health information system [38], although the local DOH had developed their own solution called Catch & Match. Health information systems can be utilized in the COPC approach to contribute to analysis of local health needs and assets (community diagnosis), prioritization of health needs, and development of interventions in an evidence-based and scientific decision making process [12]. Although CHWs collected data from household assessments and support groups this was paper-based, and not analysed or integrated into the health information system [39]. The health information system was not fit for purpose and only monitored the performance of the CHWs. Although mHealth technology appears a suitable solution for CHWs there are concerns with safety and crime, and implementation needs to be carefully planned to be successful [40]. Collecting data on NCDs can enhance the commitment of CHWs to NCD prevention [23].

CHWs had to share kitbags within their groups because they often lacked equipment such as a variety of blood pressure cuff sizes, scales, or patient information leaflets. Other studies have also found that CHWs are handicapped by a lack of essential equipment and the budget commitment to provide such equipment [12, 19, 21]. These CHWs were fairly well equipped and had access to essentials such as uniforms, transport, and stationary, but needed additional equipment for some of their specific activities.

### Limitations

As with all qualitative research the findings are highly contextual, however many of the findings could be transferred to similar settings in the Western Cape or South Africa where CHWs are trying to provide a service for chronic diseases. Although the data was collected pre-COVID-19 in 2018 the disruption to COPC caused by COVID-19 means that most of these findings are still relevant to the implementation of community-based services.

Strengths of the study included the triangulation of data sources (CHWs and managers from NGO and DOH) and data types (interviews and observations) and the assessment that data saturation was achieved when no new themes emerged from the interviews. Although data collection and analysis was performed by DW, the process was supervised by RM.

The responses and willingness of the CHWs to participate in the research could have been influenced by perceived hierarchy and power dynamics between the researcher and the participants. Awareness of the researcher’s identity and being affiliated with the district hospital, could have made CHWs believe they were being assessed. In an attempt to empower CHWs in the interview a copy of the interview guide was given to the participants beforehand and interviewees could speak their preferred language (English or Afrikaans). CHWs were not familiar with the researcher, and she had no supervisory or formal role in their work. CHWs were also interviewed at a time and place of their convenience.

### Recommendations

Based on the findings of this study several recommendations can be made:

- CHWs can contribute to the prevention of NCDs through health promotion targeting the underlying risk factors. This could happen during household visits and support groups, as well as in other social, working and learning spaces in the community [7]. They will need skills in behaviour change conversations as well as knowledge of lifestyle modification. Attention should also be given to their own risk factors and lifestyle.
- CHWs can assist with screening for risk factors and early diagnosis. CHWs are able to assess non-laboratory based cardiovascular disease risk [41]. They can also refer people for screening and further assessment, such as for cervical cancer. Screening for hypertension and diabetes is possible but requires the necessary equipment and training.
- CHWs can assist with adherence support and delivery of medication to people with known NCDs. They can also help with linkage to care for those that are lost to follow up, are uncontrolled or have complications.
- Their roles in rehabilitation and palliative care need further definition and training. The nurse coordinator may have a clinical role in home-based assessment and care planning.
- All of their roles should be supported by clear policy, guidelines and standardised training. Supportive supervision is essential, and nurses need to have the capacity to fulfil this role. The ratio of professional nurse coordinators to CHWs should be improved. Coordination and functional integration between the facility-based and community-based members of the PHC team is also required. Relationships need to be built on mutual respect, understanding and regular multidisciplinary engagement. Referral pathways should be strengthened.
- Inter-sectoral collaboration should be strengthened to support the work of the CHWs. This should enable appropriate referrals to governmental, non-governmental and private services and support both health and social care.
- CHWs can provide useful information on community health needs, particularly through household assessment and registration. However, data needs to be captured more efficiently and electronically, analysed to provide information, and integrated with other data from facilities.
- The COPC approach needs to be fully implemented as it provides a model of care within which CHWs can function effectively.

## Conclusion

The CHWs displayed a strong sense of significance and pride in their work because they were embedded in the communities they served. Their role was potentially comprehensive, but limited by a lack of sufficient training, inadequate supportive supervision, poor inter-sectoral support from social services and a need for more clarity on their roles in rehabilitation and palliative care. Training might also have been limited by low educational backgrounds.

A number of opportunities and threats were identified such as poor remuneration and labour law issues, poor integration of community- and facility-based teams, the need for a more functional and electronic data collection system that was linked to the district health information system, and some deficiencies in terms of equipment and resources.

### Electronic supplementary material

Below is the link to the electronic supplementary material.

Supplementary Material 1

## Data Availability

The data that support the findings of this study are not openly available as participants did not consent to their qualitative data being shared in this way, but data may be available from the corresponding author upon reasonable request.

## Abbreviations

BP: Blood pressure
COPC: Community-orientated primary care
COVID-19: Corona virus infectious disease 2019
DOH: Department of health
HIV: Human immunodeficiency virus
MHS: Metro Health Services
NCD: Non-communicable diseases
NGO: Non-government organisation
PHC: Primary health care
TB: Tuberculosis
WBOT: Ward based outreach team

## References

[1] National Department of Health. National Strategic Plan for the Prevention and Control of Non-Communicable Diseases, 2022–2027 [Internet]. Pretoria, South Africa. 2022. https://www.sancda.org.za/wp-content/uploads/2022/06/NCD-NSP-Final-For-Circulation_June-2022.pdf.

[2] Statistics South Africa (2020). Statistical release mortality and causes of death in South Africa 2017: findings from death notification. Stat Release.

[3] Mash RJ, Presence-Vollenhoven M, Adeniji A, Christoffels R, Doubell K, Eksteen L et al. Evaluation of patient characteristics, management and outcomes for COVID-19 at district hospitals in the Western Cape, South Africa: Descriptive observational study. BMJ Open [Internet]. 2021 Jan 26 [cited 2021 Apr 22];11(1). https://pubmed.ncbi.nlm.nih.gov/33500292/. 10.1136/bmjopen-2020-047016 PMC7839306 33500292

[4] Boake M, Mash R (2022). Diabetes in the Western Cape, South Africa: a secondary analysis of the diabetes cascade database 2015–2020. Prim Care Diabetes.

[5] Matsoso M, Fryatt R, Andrews G. The South African Health Reforms, 2009–2014: Moving Towards Universal Coverage [Internet]. Cape Town: Juta; 2015 [cited 2018 Dec 18]. https://scholar.google.co.za/scholar?hl=en&as_sdt=0%2C5&q=South-African-Health-Reforms-2009-2014&btnG=.

[6] Daviaud E, Besada D (2017). Saving lives, saving costs: Investment Case for Community Health Workers in South Africa.

[7] Mash R, Goliath C, Mohamed H, Reid S, Hellenberg D, Perez G (2020). A framework for implementation of community-orientated primary care in the Metro Health Services, Cape Town, South Africa. Afr J Prm Heal Care Fam Med.

[8] Van Rensburg H, Van Rensburg H, editors. Pretoria: Van Schaik; 2012.

[9] Western Cape Government. A framework for introduction of differentiated models of care (Circular H49/2023). Cape Town; 2023.

[10] Census. 2011 [Internet]. 2011 [cited 2020 Jun 9]. City of Cape Town. http://www.statssa.gov.za/?page_id=993&id=city-of-cape-town-municipality

[11] Masincedane Community Service [Internet]. 2024 [cited 2024 Jan 23]. https://www.masincedane.com/about_us.

[12] Mash B, Ray S, Essuman A, Burgueño E (2019). Community-orientated primary care: a scoping review of different models, and their effectiveness and feasibility in sub-saharan Africa. BMJ Glob Heal.

[13] Western Cape Government Health. Healthcare 2030. The road to wellness. Cape Town; 2014.

[14] Government of South Africa. 1. Chapter 10: Promoting Health. In: National Development Plan 2030. 1st ed. Pretoria: Government of South Africa. 2013. pp. 330–51.

[15] Ritchie J, Spencer L, Bryman A, Burgess R (1994). Qualitative data analysis for applied policy research. Qualitative data analysis.

[16] National Planning Commision (2012). National development plan 2030: our future - make it work. The Presidency.

[17] Stephens JH, Addepalli A, Chaudhuri S, Niyonzima A, Musominali S, Uwamungu JC et al. Chronic disease in the community (CDCom) program: hypertension and noncommunicable disease care by village health workers in rural Uganda. PLoS ONE. 2021;16(2 February). 10.1371/journal.pone.0247464 PMC7906377 33630935

[18] Ajisegiri WS, Abimbola S, Tesema AG, Odusanya OO, Peiris D, Joshi R. We just have to help: Community health workers’ informal task-shifting and task-sharing practices for hypertension and diabetes care in Nigeria. Front Public Heal. 2023;11. 10.3389/fpubh.2023.1038062 PMC9909193 36778542

[19] Rawal LB, Kharel C, Yadav UN, Kanda K, Biswas T, Vandelanotte C et al. Community health workers for non-communicable disease prevention and control in Nepal: a qualitative study. BMJ Open. 2020;10(12). 10.1136/bmjopen-2020-040350 PMC7737104 33318116

[20] Chen J, Yu G, Li W, Yang C, Ye X, Wu D et al. A situational analysis of human resource and non-communicable diseases management for community health workers in Chengdu, China: a cross-sectional study. BMC Health Serv Res. 2023;23(1). 10.1186/s12913-023-09880-z PMC10576308 37833662

[21] Rawal L, Jubayer S, Choudhury SR, Islam SMS, Abdullah AS. Community health workers for non-communicable diseases prevention and control in Bangladesh: a qualitative study. Glob Heal Res Policy. 2021;6(1). 10.1186/s41256-020-00182-z PMC7786185 33407942

[22] Musoke D, Atusingwize E, Ikhile D, Nalinya S, Ssemugabo C, Lubega GB et al. Community health workers’ involvement in the prevention and control of non-communicable diseases in Wakiso District, Uganda. Global Health. 2021;17(1). 10.1186/s12992-020-00653-5 PMC7791672 33413500

[23] Tesema AG, Peiris D, Joshi R, Abimbola S, Fentaye FW, Teklu AM et al. Exploring complementary and competitive relations between non-communicable disease services and other health extension programme services in Ethiopia: a multilevel analysis. BMJ Glob Heal [Internet]. 2022 Jun 1 [cited 2024 Jan 26];7(6). http://www.ncbi.nlm.nih.gov/pubmed/35738842. 10.1136/bmjgh-2022-009025 PMC9226884 35738842

[24] Donovan J, O’Donovan C, Kuhn I, Sachs S, Winters N. Ongoing training of community health workers in low-income and middle-income countries: A systematic scoping review of the literature. BMJ Open [Internet]. 2018;8(4):e021467. https://www.ncbi.nlm.nih.gov/pubmed/29705769. 10.1136/bmjopen-2017-021467 PMC5931295 29705769

[25] Van Rensburg M, Marcus T. Evaluating community health worker education policy through a national certificate (vocational) primary health qualification lens. Afr J Prm Heal Care Fam Med [Internet]. 2020;12(1):a2104. https://phcfm.org/index.php/phcfm/article/view/2104/3577. 10.4102/phcfm.v12i1.2104 PMC7061226 32129653

[26] Malan Z, Mash B, Everett-murphy K. A situational analysis of training for behaviour change counselling for primary care providers, South Africa. Afr J Prm heal care. Fam Med. 2015;7. 10.4102/phcfm.v7i1.731 PMC4564904 26245589

[27] Yenit MK, Kolbe-Alexander TL, Gelaye KA, Gezie LD, Tesema GA, Abebe SM et al. An Evaluation of Community Health Workers’ knowledge, attitude and personal lifestyle Behaviour in Non-communicable Disease Health Promotion and Their Association with self-efficacy and NCD-Risk perception. Int J Environ Res Public Health. 2023;20(9). 10.3390/ijerph20095642 PMC10178727 37174162

[28] Scheffler E, Mash R (2023). Evaluation of a stroke rehabilitation training programme for communitybased primary healthcare. Afr J Disabil.

[29] Scheffler ES, Mash R (2020). Figuring it out by yourself: perceptions of homebased care of stroke survivors, family caregivers and community health workers in a low-resourced setting.

[30] Scheffler E, Mash R. Surviving a stroke in South Africa: outcomes of home-based care in a low-resource rural setting. Top Stroke Rehabil. 2019;26(6). 10.1080/10749357.2019.1623473 31169468

[31] Van Heerden E, Jenkins L (2022). The role of community health workers in palliative care in a rural subdistrict in South Africa. Afr J Prm Heal Care Fam Med.

[32] National Department of Health. Policy and Framework and strategy for Ward Based Primary Healthcare Outreach Teams [Internet]. 2018 [cited 2024 Jan 23]. https://www.health.gov.za/wp-content/uploads/2020/11/policy-wbphcot-4-april-2018_final-copy.pdf.

[33] Lucas J, Sustainability. and financial implications of the Labour Relations Amendment Act No.6 of 2014 for Western Cape health services outsourcing [Internet]. Stellenbosch University; 2017. https://scholar.sun.ac.za/items/4d4fb316-621b-4abd-9df6-f4bff1f42f14.

[34] Zulu J, Kinsman J, Michelo C, Hurtig A. Integrating national community-based health worker programmes into health systems: A systematic review identifying lessons learned from low-and middle-income countries. BMC Public Health [Internet]. 2014;14(987):1–17. http://www.biomedcentral.com/1471-2458/14/987. 10.1186/1471-2458-14-987 PMC4192351 25245825

[35] Brey Z, Mash R, Goliath C, Roman D. Home delivery of medication during Coronavirus disease 2019, Cape Town, South Africa: Short report. African J Prim Heal Care Fam Med [Internet]. 2020 Jun 4 [cited 2020 Jun 6];12(1):4. https://phcfm.org/index.php/phcfm/article/view/2449. 10.4102/phcfm.v12i1.2449 PMC7284162 32501022

[36] Porter JD, Mash R, Preiser W. Turnaround times – the Achilles’ heel of community screening and testing in Cape Town, South Africa: A short report. African J Prim Heal Care Fam Med [Internet]. 2020 Oct 2 [cited 2020 Nov 5];12(1):3. https://phcfm.org/index.php/phcfm/article/view/2624/4257. 10.4102/phcfm.v12i1.2624 PMC7564763 33054266

[37] Mash R, Goliath C, Perez G. Re-organising primary health care to respond to the Coronavirus epidemic in Cape Town, South Africa. African J Prim Heal Care Fam Med [Internet]. 2020 Nov 5 [cited 2020 Nov 5];12(1):4. https://phcfm.org/index.php/phcfm/article/view/2607/4263. 10.4102/phcfm.v12i1.2607 PMC7669993 33181873

[38] National Department of Health. mhealth strategy (2015–2019) South Africa [Internet]. Pretoria. 2015. https://www.hst.org.za/publications/NonHSTPublications/mHealth Strategy 2015.pdf.

[39] Mash R, Du Pisanie L, Swart C, van der Merwe E. Evaluation of household assessment data collected by community health workers in Cape Town, South Africa. South African Fam Pract [Internet]. 2020 Dec 3 [cited 2021 Jan 28];62(1):6. https://safpj.co.za/index.php/safpj/article/view/5168. 10.4102/safp.v62i1.5168 PMC8378136 33314942

[40] Greenhalgh T, Abimbola S. The NASSS framework-a synthesis of multiple theories of technology implementation. Appl Interdiscip Theory Heal Informatics [Internet]. 2019 [cited 2022 Feb 20];263:193–204. https://books.google.co.za/books?hl=en&lr=lang_en&id=72OwDwAAQBAJ&oi=fnd&pg=PA193&dq=The+NASSS+Framework+–+A+Synthesis+of+&ots=hslbd8jrm7&sig=oUp75xHbBwim0Wi0rD6S-PDe4m4 10.3233/SHTI190123 31411163

[41] Gaziano TA, Abrahams-Gessel S, Denman CA, Montano CM, Khanam M, Puoane T et al. An assessment of community health workers’ ability to screen for cardiovascular disease risk with a simple, non-invasive risk assessment instrument in Bangladesh, Guatemala, Mexico, and South Africa: an observational study. Lancet Glob Heal [Internet]. 2015 Sep [cited 2020 Jun 9];3(9):e556-63. http://www.ncbi.nlm.nih.gov/pubmed/26187361. 10.1016/S2214-109X(15)00143-6 PMC4795807 26187361

