# Supplementary material for: The role of community health workers in non-communicable diseases in Cape Town, South Africa: descriptive exploratory qualitative study
Source: BMC Prim Care. 2024 May 21;25:176. doi: 10.1186/s12875-024-02424-2 (PMC11106914; doi:10.1186/s12875-024-02424-2)
Supplement: Supplementary file 1 — Supplementary Material 1 [file 12875_2024_2424_MOESM1_ESM.pdf]

Appendix B

Community health worker interview guide

Opening questions

What is your role as a community health worker in the management of chronic diseases?

What role do CHWs have with the identification and treatment of the risk factors for chronic diseases?

Other topics

a. Their relationship with the community
    1. Can you describe the relationship that community health workers have with the community?
    2. Why is it important for community health workers to have such a relationship with the community?
    3. How does the relationship that community health workers have with the community assist in your daily work activities / chronic diseases?

b. The strengths and weaknesses of the current services for chronic diseases

    1. How do the current services improve the management of chronic diseases?

    2. In what way should the current services be improved in order to treat patients with chronic diseases better?

c. Cooperation, referral to and partnership with primary care facilities and staff
    1. How do you experience your referral of patients to the primary care facilities and clinics?
    2. Tell me about your relationship with the staff at the primary care facilities?

d. Pros and cons of their current training in NCDs
    1. How does your training help you in treating patients with chronic diseases?
    3. In what ways should your training be improved to assist you with treating patients with chronic diseases?

e. Their perceptions of the future COPC model for CHWs
    1. What are your views of the proposed COPC model?
    2. How do you find the ‘catch and match” mhealth technology

<u>NGO manager interview guide</u>

Opening questions

- How is the relationship of the NGO/CHWs with the community?
- What services does the NGO/CHWs render in the management of chronic diseases?

Further topics

The strengths and weaknesses of the current services for NCDs

- How can the current management of chronic diseases be improved?
- What are the strengths and weakness of the current services for the management of chronic diseases?

Cooperation, referral to and partnership with primary care facilities and staff

- How do you experience the referral of patients to primary health care facilities?
- How is the relationship between the NGO (CHWs) and the primary care facilities?

Pros and cons of their current training in NCDs

- How do you think the training programme should be improved in order to manage chronic diseases more effectively?

Inter-sectoral collaboration

- How does partnership with other sectors impact on the services the NGO delivers?

Relationship with the department of health

- Describe your relationship with the department of health

Monitoring and evaluation of the CHWs and health information systems

- Can you explain how community health workers are supervised
- How is health information collected and processed
- Can you describe how patients are referred to and from community based services
- How is health information of patients used in clinical audits of disease and is it used by primary care facilities

Their perceptions of the future COPC model for CHWs

- What are your views on the future COPC model in the pilot sites
- How well is the 'catch and match' mhealth technology working

Supervision and support

- How often do you meet with CHWs to discuss work planned
- How are CHW supervised and supported when doing household visit

<u>Interview guide for sub-district manager</u>

Opening question

What do you think are the strengths and weaknesses of the current service by CHWS for NCDs?

Further topics

a. Relationship with the NGO and CHWs
   Can you describe your current relationship with the NGO/CHWs in the sub-district?

b. Monitoring and evaluation of the CHWs and health information systems
   What is your role in the evaluation of community health workers and health system surveillance pertaining to the information collected from community health workers?

c. Assessment of their current impact
   How is the current impact of community health workers measured?
   What is the current impact of community health workers on the health system?
   What are the expected impact and role that community health workers have?

d. Strengths and weaknesses of the current services for NCDs
   What do you think are the strengths and weaknesses of the current service for NCDs?

e. Perceived roles of the CHWs in relation to NCDs now and in the future
   What is your view of the COPC model for community-based services
   What is the role of the CHW in relation to NCDs in the COPC programme?

f. Training needs and current training cost-effectiveness and financial issues
   What are some of the financial issues pertaining to CHW employment with-in the sub-district?
   What are the training needs of community health workers that the department of health identifies?
